# TuberOus SClerosis Registry to Increase Disease Awareness: A Review on Alignment of Its Planning, Execution, and Publications With European Medicines Agency Guidelines

**DOI:** 10.3389/fneur.2020.00365

**Published:** 2020-05-15

**Authors:** Ruben Marques, Henriette Thole, José G. Ruiz de Morales

**Affiliations:** ^1^Institute of Biomedicine (IBIOMED), University of Leon, León, Spain; ^2^Real World Evidence Centre of Excellence Oncology, Novartis Pharma AG, Basel, Switzerland

**Keywords:** tuberous sclerosis complex, rare disease, rare disease registry, patient registry, tuberous sclerosis registry to increase disease awareness

## Abstract

Patient registries offer a powerful and practical means of real-world data collection system for rare diseases. Many guidelines have been released to standardize patient registries, although most of them do not address issues specific to rare disease patient registries. In November 2018, the European Medicines Agency (EMA) released a draft discussion paper on methodological and operational aspects of disease registries and made proposals on good registry practice (henceforth referred to as EMA guidance). This guidance was highly anticipated by all stakeholders with a strong interest toward governance, operationalization, and study conduct in registries. With improved clarity toward conduct of patient registries, this guidance will encourage overall registry use in regulatory decision making. TuberOus SClerosis registry to increase disease Awareness (TOSCA) was an international, multicenter patient registry to assess the manifestations, interventions, and outcomes in patients with tuberous sclerosis complex (TSC). The planning of TOSCA was initiated in 2011, patient enrolment commenced in August 2012, and final analysis database was locked in August 2017, long before the EMA guidance was released. Moreover, initial publications of TOSCA, such as first interim analysis, had also been published before the release of the EMA guidance. Extensive feedback and lessons learned from the TOSCA registry have provided insights into rare disease registry planning and operations. In this paper, we tested the recommendations from the EMA guidance on a rare disease registry, that is, the TOSCA registry. We elaborated the compliance and deviations of the TOSCA registry from the EMA guidance on a point-by-point basis. A careful observation revealed that in most aspects, TOSCA was in compliance with EMA. However, there were several practical issues identified in TOSCA, which deviated from EMA guidance. These issues demonstrate that deviations from EMA guidance, particularly in rare disease registries, do not signify compromised registry quality and can be somewhat expected in small populations. Despite multiple deviations of TOSCA from the EMA guidance, TOSCA was able to meet its objectives to enhance our understanding of TSC and its manifestations.

## Introduction

### Role of Patient Registries in Rare Diseases

Rare diseases, owing to the limited number of patients and phenotype diversity, often lack a thorough research in terms of underlying pathology of the disease, as well as the course of disease, its manifestations, and the outcomes (1, 2). Although the impact of an individual rare disease may appear limited, the collective burden of rare diseases on public health is enormous. Moreover, the awareness and knowledge about rare diseases among primary care physicians is limited.

The real-world data (RWD) collected in patient registries offer valuable insights on the disease itself, the effectiveness, and safety of particular therapies and play a crucial role in health-care decision making (1). Patient registries aid the understanding of natural history, evolution, risk, and outcomes of specific diseases. They support the research on genetic, molecular, and physiological bases of rare diseases. Furthermore, rare disease registries often fill a social gap as well, by connecting patients and families who are facing similar challenges as well as clinicians working in the same disease area. They may also establish a patient base for the evaluation of drugs, medical devices, and orphan products and may be used as historical controls to further accelerate research in areas of high unmet medical need (3). The European Medicines Agency (EMA) frequently relies on patient registries to gather RWD on the risks and benefits of a particular product, as a condition to monitor post-marketing safety and efficacy and as a condition for approval (4). Hence, patient registries offer a powerful opportunity to further the clinical research in rare diseases and improve patient care as well as health-care planning (1).

The importance of rare disease registries has been recognized and underlined by the European Union (EU), through the “EU Council Recommendation of 8 June 2009 on an action in the field of rare diseases (5).” Through strengthening and acknowledging the valuable role of patient registries, there has been a significant boost in the number of rare disease patient registries in the recent years (6). According to the Orphanet Report Series Rare Disease Registries in Europe, May 2019, there are 69 global rare disease registries, 69 rare disease registries in Europe, and 535 rare disease registries at the national level and further at the regional level (7). However, these patient registries are diverse in terms of the objectives, patient inclusion and exclusion criteria, the core data elements, and overall data quality and completeness. Hence, for setting up a successful rare disease registry, a practical guidance with detailed consideration to all aspects of planning and execution is crucial (4). As more patient registries in rare diseases are being launched, more issues are being identified, regarding the hurdles and limitations during planning and execution of these registries. Resolving such issues and offering appropriate guidance to standardize the data elements across the registries is desired by all stakeholders and has hence received adequate emphasis in the EMA guidance.

Several efforts have been made to standardize the patient registry setting and implementation. The European Union Committee of Experts on Rare Diseases (EUCERD) adopted a set of Recommendations on Rare Disease Patient Registration and Data Collection in 2013. These recommendations formalize the consensus reached and guide all stakeholders into systematic discussions on data collection and registration (8). Furthermore, many international projects, including EPIRARE and RD-CONNECT, have been initiated to promote international registries (9). Orphanet provides direct online access to an inventory and encyclopedia of rare diseases (7). Similarly, the National Center of Rare Diseases in Italy has also released recommendations for improving the quality of rare diseases registry (6).

Patient registries are furthermore a tool frequently used in pediatric research and drug development to better understand diseases, as historical controls and as a mean to follow up patients over long periods of time. Children cannot be considered “small adults,” as age and developmental maturation vastly affect the pharmacokinetics and pharmacodynamics of many drugs. Hence, it is imperative to assess dosing, efficacy, safety, and long-term benefit/risks of any therapeutic treatment by following a dedicated pediatric drug development process, which needs careful consideration while setting up pediatric trials. Furthermore, pediatric clinical trials have to follow stricter regulations, require in-depth ethical consideration, and usually have longer follow-up periods with a smaller patient pool (10). Additionally, the need for frequent long distance travel to study sites and later switch from pediatric to adult care, including re-consent during a long-term follow-up, often results in loss of follow-up. High rates of lost follow-up in pediatric trials, such as a 55% lost follow-up in a US pediatric diabetes trial, after a median of 1.3 years from enrolment, are not uncommon (11). This makes integration of pediatric trials into routine clinical care valuable but challenging.

In an attempt to expand the overall use of patient disease registries across all populations in the benefit–risk evaluation of medicines for regulatory purposes, the EMA supports a more systematic and standardized approach to planning and execution of all patient registries. In 2015, the EMA established the Patient Registry Initiative and the Cross-Committee Task Force on registries to identify the barriers and establish good registry practices. In November 2018, the EMA issued a draft discussion paper on methodological and operational aspects of disease registries and made proposals on registry studies and good registry practice (12). In this paper, we refer to the EMA discussion paper on methodological and operational aspects of disease registries as “EMA guidance.”

The EMA guidance is a reflection of recommendations based on multiple workshops and resources, including the EMA Patient Registries Workshop, the four disease-specific workshops on registries for cystic fibrosis, multiple sclerosis, CAR-T cell products and hemophilia, the Qualification opinion on the European Cystic Fibrosis Society Patient Registry (ECFSPR), the Draft qualification opinion on the Cellular therapy module of the European Society for Blood and Marrow Transplantation (EBMT) Registry, and existing guidance published in the PARENT Joint Action Methodological Guidance and the US Agency for Healthcare Research and Quality (AHRQ)'s handbook. It is also aligned with the recommendations from the European Network of Centers for Pharmacoepidemiology and Pharmacovigilance (ENCePP) Guide on Methodological Standards in Pharmacoepidemiology and the ENCePP Code of Conduct.

The EMA guidance elaborates on multiple aspects of planning and execution of patient registries (12). Although this guidance is not specific for rare disease registries, it is expected to become the gold standard for registry guidance across all patient registries including those covering small populations, pediatric indications, and rare diseases. This shift in mindset is reflected in national health authorities enforcing the implementation of good registry practice through legal framework and national registry initiatives. For instance, the German Ministry of Health has passed the “Gesetz für mehr Sicherheit in der Arzneimittelversorgung” (13) (GSAV, Law for More Safety in the Supply of Medicines) and IQWiG (14), outlining registry use as part of the report on scientific concepts for the generation of routine practice data and their analysis for the benefit assessment of drugs.

### Overview of TuberOus SClerosis Registry to Increase Disease Awareness

Tuberous sclerosis complex (TSC) is an autosomal dominant disorder, characterized by formation of hamartomas in multiple organ systems. This rare disorder originates from genetic mutations in either *TSC1* or *TSC2* gene. In most patients, it manifests as dermatological, renal, or neurological abnormalities, although any organ system can be affected (15). This seriously debilitating disease is rare, with an estimated prevalence between 1/6,800 and 1/15,000 population. The disease is diverse in terms of age of onset, its manifestations, and its severity (16). It can be diagnosed at any point in life, even prenatally, depending on the location of tumors. The age of onset and hence diagnosis can further vary, depending on access to clinical and genetic testing. The average age of diagnosis has been reported to be around 5 years; however, it is likely that TSC is frequently underdiagnosed depending on manifestations and access to health care (17). Despite several advances made over the years, there are still gaps in the understanding of TSC. Considering the rare prevalence and diverse clinical implications, various aspects of TSC have not been documented and published adequately to assist our understanding of the condition. Moreover, many treatment options have not been monitored long term to gather high level of disease insights. This issue is also reflected in the TSC consensus panel, which acknowledged that the current TSC recommendation guidelines are not based on high levels of evidence. Hence, more information is required about TSC to improvise management strategies (16).

In order to address these existing gaps, in 2011, Novartis collaborated with medical experts and patient advocates to evaluate the need for a TSC registry. A subsequent survey highlighted that in many European countries, there were no national TSC registries or any systematic data collection for TSC. It was realized that instead of solely relying on the fragmented evidence obtained from a limited number of patients, a larger collaboration was more desirable. This consensus regarding the need to establish a TSC registry helped conceptualize TuberOus SClerosis registry to increase disease Awareness (TOSCA) (16).

Although TOSCA was initiated in Europe, some non-European countries joined the registry later, further expanding its reach. TOSCA is a multicenter, international disease registry to collect data to assess the manifestations, interventions, and their outcomes in patients with TSC. The detailed description of registry design and structure has been published earlier by Kingswood et al. (16). The baseline data of 2,093 patients in TOSCA have been already been published (18).

### Systematic Collection and Dissemination of Lessons Learned From TuberOus SClerosis Registry to Increase Disease Awareness

As TOSCA was the first multinational registry for TSC, there were various issues, predominantly in its planning and implementation. In an attempt to characterize these issues and in order to disseminate future registries in rare diseases, a questionnaire-based survey was conducted among the members of steering committee, principal investigators (PIs), and sponsor employees involved in the TOSCA registry. This survey identified key strengths and limitations regarding planning and implementation in TOSCA (19). The practical experiences in TOSCA and the lessons learned can be used to supplement the EMA guidance for future registries in rare diseases. In this paper, we refer to the TOSCA survey (19) as “TOSCA lessons paper.”

### Rationale

As stated, the drafted EMA guidance regarding good registry practice was released in November 2018; by then, the TOSCA registry was reaching the stage of final data analysis. Hence, with this paper, we strive to compare and evaluate how the TOSCA registry differs from the EMA recommendations on a point-by-point basis and whether such deviations may have affected the registry outcomes. We also analyze how the learning from TOSCA can complement the EMA guidance, especially in case of rare disease registries. The observations in this paper also incorporate the experiences and perspectives of the Clinical Trial Head (CTH) of the TOSCA registry and, hence, also offer insights regarding practical issues during the conduct of the registry.

## Observations

The suggestions derived from EMA are divided into four categories: registry planning, operations of registry, data analysis, and publication of results. The recommendations from the EMA guidance are summarized under each subheading, followed by the TOSCA methodology, along with the relevant issues, if identified, in TOSCA. The point-wise comparison and compliance of TOSCA and EMA guidance have been summarized in Table 1.

**Table 1:** Summary of TOSCA compliance with EMA guidance.

**Topic (corresponding EMA guidance chapter)**	**Recommendations from EMA guidance**	**Procedure adopted in TOSCA registry**	**TOSCA compliance with EMA guidance**
**REGISTRY PLANNING**			
Protocol preparation (5.1, 6.3)	• Meticulous predefined design and SAP in protocol • Protocol changes to be included as formal protocol amendments • Separate protocol for registry studies (e.g., PASS) • Protocol to meet ENCePP checklist	• Meticulous planning with KOLs and the other stakeholders • Six research projects included in protocol amendment • No separate protocol for registry studies (Votubia® PASS) • PASS enlisted with ENCePP	Partial
Terminologies (5.5)	• Standard Orphadata, along with ICH-9, 10 and 11, MedDRA	• MedDRA • WHO Drug Reference List, based on ATC classification system	Complete
Data collection/data elements/time elements (5.3, 5.4, 6.5)	• Wide range of data depending on registry objectives • Use “Set of common data elements for RD registration” on EURD Platform • Core list of dates to be collected	• Core (compulsory) and subsections (petals) design of data elements • Additional safety information collected for PASS • Dates collected for pre-defined relevant variables	Complete
Duration/timelines (3.3, 5.1, 6.2)	• Long-term follow-up dictated by schedules for data collection • Registry study to follow up to achieve study objective	• 5 years follow-up • Extended follow-up for PASS	Partial
**OPERATIONS OF THE REGISTRY**			
Patient enrolment (5.2, 6.4)	• Clear conceptual and operational definition of target population • Exhaustive patient enrolment • Registry study a subset of the registry population or enroll additional patients, if required	• Documented visit for TSC within the preceding 12 months or newly diagnosed • Retrospective as well as prospective data collection from 170 sites across 31 countries. • 2,214 patients enrolled in TOSCA registry, 571 in 6 RPs and 179 patients in PASS.	Complete
Informed consent (5.8.4.)	• Patients are aware: why/what data is collected, how/ by whom it will be used, and at what level of details	• Patient Information Brochure and informed consent form	Complete
Quality management (5.6, 6.6)	• Quality management inconsistency, completeness, accuracy and timelines (5.6.2, 5.6.3) • Use data quality indicators to ensure data quality (5.6.4)	• Routine measures for quality maintenance deployed on a site and registry level flagging inconsistency, completeness, accuracy. • 5 yearly interim analyses conducted to assess data quality	Partial
Data sharing (5.8.3)	• Data sharing is encouraged, at least on an aggregated and ideally on an anonymized patient-level	• Data access is enabled for investigators with specific research question, upon approval by SAB. • TOSCA investigators could request for access to self-recorded data on eCRF after the completion of registry data collection (August 2017)	Complete
Data security (5.8.5)	• Security measures should be implemented to maintain the privacy of patients	• Overseen and managed by neutral 3rd party (CRO) and clarified in contract	Complete
**DATA ANALYSIS**			
Data analysis (5.6.3, 5.7, 6.7)	• Subjective to registry purpose • Registry study to have separate SAP	• Due to exploratory registry purpose mainly descriptive analysis • PASS with yearly interim analysis but no separate SAP	Partial
Safety analysis (5.7, 6.8)	• Reporting of AEs • Monitoring of AESI • Aggregated analysis of AEs	• AE reporting at site level according to national regulations • AESI assessed in sub-population in the context of a PASS • No analysis of all AEs planned in the objectives of the registry	Partial
**PUBLICATIONS**			
Publication policy (6.9)	• Lead investigator retains authority to prepare publication of registry results. • MAH discuss final results and interpretation, if required.	• WC, with the approval of SAB developed publication strategy. • WC responsible for preparation and coordination of all presentations and publication activities. • Sponsor data owner • MAH not involved	Complete

* *Until they reach Tanner stage V or age of 16 years in females and 17 years in males*.

*ATC, Anatomic Therapeutic Classification; CRO, Clinical Research Organization; eCRF, Electronic case report forms; ENCePP, European Network of Centers for Pharmacoepidemiology and Pharmacovigilance; EURD, European Platform on Rare Diseases Registration; ICH, International Council for Harmonization; KOL, Key Opinion Leaders; MAH, Marketing Authorization Holder; MedDRA, Medical Dictionary for Regulatory Activities; PASS, Post-Authorization Safety Study; RD, Rare Diseases; RPs, Research projects; SAB, Scientific Advisory Board; SAP, Statistical Analysis Plan; TOSCA, TuberOus SClerosis registry to increase disease Awareness; WC, working Committee; WHO, World Health Organization*.

### Registry Planning

#### Design and Governance of Registry

The EMA guidance recognizes patient disease registries, particularly in rare diseases, as an important source of information derived from clinical practice. Although randomized controlled trials (RCTs) are the gold standard for gathering evidence in clinical development, patient registries are more practical and offer the best platform when conducting RCTs is not feasible or ethical, for example, when using historical control data, where comparable standard of care is lacking. It is also noteworthy that a registry is not initiated and guided by a single research question or hypothesis. Rather, it is driven with the aim to describe a disease/therapeutic treatment/patient population as a whole. The EMA guidance suggests meticulous planning, including statistical analysis plan and other details, including those for research projects. It also emphasizes the effective collaboration between all involved parties and explicitly describes the role of different stakeholders such as registry coordinators, pharmaceutical companies, and regulatory authorities (12).

Furthermore, the EMA guidance treats registry studies as a separate entity and presents a dedicated section regarding guidance for registry studies. It states that, in addition to the registry protocol, each registry study should have a stand-alone protocol with detailed description of study design, patient population, data collection, and detailed statistical analysis plan. As an aid, the EMA guidance recommends the use of the ENCePP checklist for the creation and evaluation of registry study protocols. Additionally, the protocol should follow all applicable national and regional regulations such as the Good Pharmacovigilance Practice (GVP) Module VIII, if appropriate. Any changes in either registry or study protocol should be recorded as formal protocol amendments (12).

Although TOSCA was planned and initiated much before the EMA guidance was released, all efforts were made to thoroughly plan the registry and to achieve its objectives through a systematic and reliable data collection system. The TOSCA registry organization involved key experts from different areas, including TSC medical health-care experts, representatives from pharmaceutical sponsor, as well as patient representatives in the “Scientific Advisory Board” (SAB) and “Working Committee” (WC) (16). Expert opinions and views gathered in a meeting with different stakeholders ensured careful planning of the registry prior to its launch. The SAB was responsible for the general oversight of the scientific principles and conduct of the registry and also for appropriately promoting the use of the registry in the participating sites. Furthermore, the SAB advised the WC on the implementation and development of the registry. It was also responsible to review and approve the individual research projects. The SAB furthermore covered the essential mandate on publication policy and planning. The WC was responsible for the registry content and for the coordination of all the operative activities after the registry implementation. Additionally, the WC decided on the approval/rejection of requests for registry data access from those involved in the ongoing registry study or external parties. It also reviewed the core data for quality assurance purposes, including quality control analyses.

Involvement of patient representatives was instrumental in patient enrolment and further facilitated the communication with patients. Because patient representatives generally have a better understanding of patient journey within a disease, the collaboration with patient advocacy groups significantly helped and overall facilitated the research project analyzing quality of life outcomes.

After the approval of Votubia®, the EMA requested (EMEA/H/C/002311/II/0004) a Post-Authorization Safety Study (PASS) in TSC, which was subsequently included in the TOSCA registry (16). Contrary to the recommendations of the later-released EMA guidance, the TOSCA PASS did not have a separate protocol but was incorporated in the registry protocol as a protocol amendment (refer to Table 1). The registry study protocol was furthermore listed in the ENCePP list (CRAD001MIC03-ENCePP number 3247) and The European Union electronic Register of Post-Authorisation Studies (EU PAS Register) (EUPAS3247).

The successful setup of TOSCA allowed for additional six research projects to take place in TOSCA, which were also incorporated in the registry protocol, as protocol amendments. These research projects aimed to answer certain research questions pertaining to a deeper understanding of TSC. However, in the TOSCA lessons paper, it was realized that although research projects were crucial, lack of adequate planning as well as finances for such complex projects rendered them burdensome for PIs and sponsor, which in turn, might have hampered their potential to provide new insights for different manifestations of TSC (19).

#### Registry Duration and Follow-Up

EMA acknowledges that while theoretically registries are open-ended data collection systems to gather abundant information regarding a disease and its manifestations, the practical timelines are usually dictated by financing and schedules for data collection (12). This is particularly true in rare disease and small populations, where budget restrictions usually strongly impact registry duration, registry data quality, and registry data completeness.

In the TOSCA registry, the planned duration of follow-up, once a patient was enrolled in the registry, was up to 5 years. However, in Votubia® PASS, for pediatric patients in the EU region, it was agreed to continue the follow-up till they reach Tanner stage V or until 16 years of age for females and 17 years for males. Consequently, some patients are expected to be followed up until 2027, to ensure a more thorough evaluation of long-term effect of Votubia® (16).

According to the TOSCA lessons paper, 38% participants (members of SAB, PIs, and employees of sponsor involved in registry) considered a 5-year follow-up in the main registry to be short in order to holistically assess the real-life impact of the disease. A longer follow-up would definitely be more helpful for a rare disease, especially when there are multiple manifestations (19).

### Operational Aspects

#### Patient Enrolment

While registries are prone to selection bias, pertaining to multiple confounding factors, all attempts should be made to avoid selection bias as much as possible. EMA suggests keen attention toward defining and enrolling patient population. A clear conceptual definition of target population, which can be further translated into operational definition, is suggested. Comprehensive patient enrolment requires a meticulous process to exhaustively enroll patients fulfilling the operational definition, to avoid selection bias. Voluntary and informed consent with detailed information regarding the purpose and extent of data collection, as well as its further use/sharing to external parties, is mandatory during patient enrolment. Informed consent should comply with General Data Protection Regulation (GDPR). Patients also need to be informed about their potential to restrict consent as well as their withdrawal at any time (12).

The TOSCA registry was structured to retrospectively and prospectively collect data from patients with TSC. In order to gather a large multinational cohort of TSC patients, TOSCA aimed for exhaustive recruitment, as recommended by the EMA guidance, overall enrolling 2,214 patients from 170 sites across 31 countries. Such high recruitment rates, particularly for a rare and predominantly pediatric disease registry like TOSCA, is commendable. This may only have been achieved through the close collaboration with all stakeholders as well as using the recommended clear conceptual and operational definition of target population. Aligned with the EMA recommendations (refer Table 1), all patients who are enrolled in TOSCA signed a voluntary informed consent form. Separate informed consent forms were issued for research projects as well as PASS study (16, 18).

#### Site/Database Management and Quality Control

Frequently, uncertainties in data quality impact the confidence in validity and reliability of data quality in registries. Such issues are particularly critical for post-authorization registry studies, where data quality may have a significant impact on marketing authorization. EMA suggests four main activities for quality management, namely, quality planning, quality assurance, quality control, and quality improvement. Maintaining data quality comprises four major components: data consistency, data completeness, data accuracy, and data timelines. Measures to continuously assure data quality should be in place at management level as well as operational level of the registry. The EMA guidance also suggests using indicators of data quality to regularly measure and improve data quality (12).

In TOSCA, suitable measures were taken for adequate site management and data quality. Before site activation, the participating personnel at registry sites underwent thorough training and detailed protocol review with designated representatives from Novartis to ensure high data quality. Only trained and designated registry staff were allowed data entry into the Novartis-provided electronic case report form, using fully validated software that complied with the regulatory requirements for electronic data capture. Additionally, the international clinical research organization responsible for management of the web-based system was also responsible for reviewing the collected data for completeness and accuracy. Online validation checks minimized data entry errors and hence any queries. The physicians participating in the registry were responsible for ensuring timely and accurate data collection. Quality assurance reviews, audits, and evaluation of registry progress were conducted at regular intervals by authorized representatives from Novartis and regulatory agencies.

Although there were no specific data quality indicators used (refer to Table 1), maintenance of data quality and accuracy was evaluated in the first administrative analysis of the registry data. This included the data for the first 100 patients, where a total of 469 fields of information were evaluated for each of the 100 patients. In more than 90% of patients, the information on at least 85% of the fields was found to be complete. This analysis demonstrated a high degree of accuracy, hence ensuring optimum quality of data collection (16). In total, five annual interim analysis were conducted. During further planned annual interim analyses for data quality, any inconsistencies, if found, were traced back to the source site, and adequate measures were taken for its in-site modification.

In the TOSCA lessons paper, 25% of the respondents had concerns regarding the presence of some form of bias, which may be selection bias, information bias (subjected to selective recall and inconsistent data collection), or measurement bias (misclassification of outcomes). These biases may have compromised the validity of collected data. It was recommended that further efforts must be made to minimize biases, which are particularly likely to occur in registries and, further, more likely in a rare disease setting. Involvement of a statistician from the planning stage itself may help minimize the potential for biases in future registries (19).

### Data Handling

#### Data Elements

The EMA guidance suggests the use of harmonized core data and core time elements collected in a predefined format across all patient registries for the same disease to assure interoperability and comparability. Harmonization to international standards further facilitates the implementation of a common data quality system, data exchange, and further interpretation and comparison of results from different registries. Lack of harmonization leads to a time-intensive and resource-intensive process, when mapping data elements of multiple sources (12).

A list of core data elements and corresponding dates is ideally composed of “crucial” and “should have” data elements. The crucial data elements are defined as those important data and time elements that have to be collected in all registries and hence require greater resource allocation to ensure completeness, standardization, data quality, and verification of the information. The “should have” data and time elements are additional data and time elements, which are of interest and important for some stakeholders or in some subpopulation, but not essential to all (12).

Core data and time elements for a particular registry should be identified with intensive discussions among clinicians, disease experts, patient representatives, and, if required, regulatory authorities. A standard set of core data elements for rare diseases has been developed as “Set of common data elements for RD registration” on the European Platform on Rare Diseases Registration (EU RD Platform) (20). Furthermore, some disease-specific lists of core data elements are available, for example, those for cystic fibrosis (21), multiple sclerosis (22), CAR-T cell products (23), and hemophilia (24), and have been agreed upon at multi-stakeholder workshops organized and published through the EMA.

The details pertaining to the data and time elements in the TOSCA registry have already been published earlier (16). In brief, TOSCA followed a flower-and-petal model of data elements. The main “core” section was designed to collect a general predefined set of patient background data including demographics, family history, prenatal history, and disease features (i.e., neurological, neuropsychiatric, renal, cardiovascular, and pulmonary) including the corresponding dates, where relevant. This mandatory section ensured that at least a minimum amount of essential information on each patient was collected across all countries to allow meaningful analyses. Additional and more detailed data related to specific disease manifestations were collected in the “petal segments,” that is, subsections of the registry that may have only taken place in certain countries, sites, or subpopulations.

Furthermore, it is to be noted that the data elements used in TOSCA registry may form a sample list of identified data elements for future registries in TSC, especially when unlike cystic fibrosis, there is a lack of standard set of core data elements in TSC.

#### Terminologies

In order to internationally harmonize various registries across same diseases, it is recommended to use international terminologies for diseases, diagnostic tests, symptoms, medicinal products, and adverse events (AEs). When national or local terminologies are used, mapping to international terminologies is recommended (12).

The EMA guidance recommends use of standard Orphadata (25) for terminologies associated with rare diseases, along with ICH-9, 10, and 11 and Medical Dictionary for Regulatory Activities (MedDRA) (26) for standardizing terminologies. MedDRA is also internationally acceptable for AE classification for regulatory purposes.

As per the TOSCA protocol, medical history/current medical conditions were coded using the MedDRA (26). Additionally, the World Health Organization (WHO) Drug Reference List (27), which employs the Anatomical Therapeutic Chemical (ATC) classification system, was used to code the concomitant medications.

#### Data Analysis

EMA suggests using appropriate statistical method to justify the individual research question and variables in individual registry. Data analysis should be performed based on predefined time schedules. The handling of missing data should be described in the statistical analysis plan. The statistical plan for registry study should be different from the registry itself. Hence, a clearly defined statistical analysis plan for the registry studies should be provided and may be stand-alone or elaborated in detail as part of the registry study protocol. Furthermore, any changes in the statistical analysis plan should be recorded as formal protocol amendments (12).

As a part of the data analysis, the EMA guidance suggests the reporting of AEs, the monitoring of AEs of special interest, and the aggregated analysis of AEs. It is, however, to be noted that in multinational registries, following the local requirements on AE reporting is essential. Hence, in TOSCA, various sites reported the AEs to their corresponding national authorities. The AEs of special interest were predefined and assessed as a part of Votubia® PASS in the specifically described subpopulation. Because the objective of TOSCA was inclined toward describing the multitude of TSC manifestations, a detailed analysis of reported AEs was not attempted. However, specific AEs may be analyzed in the context of individual patient subgroups and contextualized with a particular manifestation.

Considering the exploratory nature of the TOSCA registry, and in the absence of a specific hypothesis put to test, the demographic and clinical parameters underwent descriptive analysis for relevant variables. Furthermore, missing data were not imputed, in general. For partially missing data, the values were imputed for analysis purpose. For example, in a renal angiomyolipoma patient, whose data regarding diagnosis and epidemiology are available but treatment details were missing, the patient's data was included in the analysis.

In the TOSCA lessons paper, 32% of respondents had concerns related to the handling of missing data. In fact, a major challenge for the TOSCA registry was to ensure that data about all the disease manifestations, for each patient, were reported, even though the different sites involved did not always follow patients for all disease manifestations in the same way, as part of routine clinical care. Noteworthy is that variables with the most missing data were related to a particular manifestation, that is, TSC-associated neuropsychiatric disorders (TAND). This may be attributed to the lack of knowledge of TAND-related manifestations investigated through the physician-reported or patient/caregiver-reported outcomes. For other manifestations, the missing data were minimal, reflecting an overall good quality data collection (19).

Although there was no definitive statistical analysis plan, adequate attempts were made to open-endedly analyze and interpret data and identify any potential correlations. Further data analysis during manuscript preparation ensured the identification of interesting insights regarding different manifestations of TSC.

#### Data Ownership and Data Sharing

EMA guidance clearly states that the control on the use of data lies with the patients, who may decide to consent or not consent for the use of their data for clinical or research purpose and may also withdraw the previous consent.

EMA guidance dictates that the registry centers and coordinators should ensure the use and sharing of data in accordance with the EU GDPR and the patient-signed informed consent form. When contractual sharing of data with Marketing Authorisation Holder (MAH) is required, the agreement should clearly describe the extent of data access, the intellectual property rights arising from the data usage, and results dissemination.

As EMA guidance suggests, all patients, before their enrolment in TOSCA, were informed about their rights regarding the generation and usage of their data. Consequently, separate informed consent forms were signed for inclusion into main registry, PASS, and individual research projects. Hence, patients had a control for the use of their data in individual studies. They were also informed about their right to withdraw consent at any time.

Members of SAB and WC had access to the consolidated and detailed data along with the results of every interim analysis. Furthermore, appropriate data access was given to investigators who submitted a research request after endorsement by the SAB. For such purposes, a contract stating the extent of data access and intellectual property rights arising from use of data was signed to avoid any conflicts. PIs had also access to self-recorded data after the completion of data collection (i.e., August 2017). The final ownership of data generated in the registry was with the sponsor.

### Publication

EMA states that regardless of the funding source, the lead investigator retains primary authority to independently prepare publications of the study results. If applicable, the MAH co-funding the registry study is entitled to view the final results and interpretations prior to submission for publication. The MAH may also share their views regarding the study results and interpretation, in advance of submission within a reasonable time limit, for example, 1 month, and without unjustly delaying the publication. EMA also entitles the MAH to request change in presentation of results to delete confidential information (12).

Because TOSCA was not aimed for a drug dossier submission approval, the MAH did not participate in the publication process. Instead, only the Novartis medical department (medical affairs) was involved in publication preparation and review.

In the initial stages of the registry, the publication policy was not well-defined. After the first manuscript, the need for a thorough publication policy and plan was realized, and the issue was rectified through a detailed publication policy released in January 2015. The WC, in turn, was responsible to develop publication strategy, which was further approved by SAB. The WC was further deemed responsible for the development and coordination of presentations and publications activities according to the publication policy. This publication policy and the planned information dissemination were clearly in line with the EMA guidance and contributed to the increased awareness of TSC.

The publication policy stated that at least one manuscript would be published following each interim analysis. Secondary manuscripts and abstracts to publications were planned to communicate the results and knowledge to a wider audience. In a further attempt to reach a broader audience, translations of posters presented at International Congresses were encouraged to be presented in local languages at National Congresses. This extension of audience reached complemented the primary objective of TOSCA: to increase awareness about this rare disease and its manifestations. A clear protocol was prepared with regard to the process of developing presentations and publications. A kick-off meeting (face-to-face or teleconference) with all authors and reviewers was suggested to discuss all details, that is, timelines, journal, and relevant topics regarding the manuscript before the initiation of manuscript writing. SAB retained the final authority regarding authorship and order or authorship.

The results of the TOSCA registry analyses were presented as posters/presentations on the main TSC, or specific manifestations, congresses. So far, nine publications from the TOSCA registry study have been released (16, 18, 19, 28–33), including its methodology, baseline analysis from second interim analysis, epilepsy, renal angiomyolipoma, subependymal giant cell astrocytoma (SEGA), and TAND from third interim analysis, SEGA in adults from final analysis, treatment patterns, and use of resources in TOSCA and learning from TOSCA. A robust publication plan for data derived from the main registry as well as research projects and the TOSCA PASS study is in place, and it is expected to be achieved by 2020. Furthermore, 15 oral presentations and 27 posters have been presented at International Congresses. Of these, five oral presentations and eight posters have been further translated and presented in National and Local Congresses. Additionally, three posters with country-specific data have been presented at National Congresses. In the future, data collected in TOSCA may be used for performing new analysis to address specific research questions on the basis of retrospective observations. In-depth analysis of specific data will further help the clinicians to have a better understanding of TSC and its manifestations.

## Sustainability

EMA recognizes that most patient registries face sustainability issues after the initial phase of funding for initiation of registry. Throughout the registry duration, sustainable funding is required for multiple reasons including maintenance of core registry features, adaption to changes in legal requirements, additional staff hiring for specific studies, and provision of funds to local centers, as necessary. In a Patient Registry Workshop, EMA recommended to consider the learning from existing successful registries to inform the sustainability component in the planning of new registries. Registry holders should engage with public agencies and define/clarify the long-term role of industry, instead of aiming for a short-term funding support. A clear development strategy, appropriate management, and the clear stakeholder partnership may help improve sustainability (34). Furthermore, EMA suggests the collaborations to have cost-sharing agreement, indicating that a registry be co-founded by multiple partners and coordinated through an “independent third party,” for example, a disease association.

The TOSCA registry was solely sponsored by Novartis, and the budget was ensured at the stage of planning of the registry. Even after the completion of data collection in the main registry in August 2017, the publication plan is being implemented with Novartis sponsorship.

With the initial registry planning, no funding issues were expected. However, six research projects were added later as protocol amendment. These research projects lacked adequate time and resource planning and had budget constraints, as they were not of primary interest in the context of any compound. Despite these issues, the research projects were able to capture important information regarding the diverse manifestations of TSC, which will enhance the understanding about the disease and its manifestations. Including research projects at the registry planning stage would ensure a more robust data collection and also improve the outcomes achieved.

## Conclusion

Comparing the EMA guidance on Good Registry Practice with TOSCA protocol and implementation course, it appears that TOSCA did not completely comply with all aspects of the EMA guidance (refer to Table 1). However, on most important aspects, the TOSCA registry is definitely in accordance with the EMA guidance. This is especially noticeable on the meticulous planning with involvement of multiple stakeholders, careful implementation ensuring valuable and high-quality data collection, definition of core and extended data elements, inclusion of research projects, and registry studies. Hence, despite partial compliance and multiple deviations from EMA guidance, TOSCA was able to successfully achieve the desired outcomes and fulfill its objectives, particularly in improving our understanding about TSC and its manifestations, as well as increasing the awareness about this rare disease. It is furthermore particularly commendable that the TOSCA registry managed to recruit such a large number of patients across all geographic regions, which would not have been possible without such a strong collaboration between stakeholders. More compliance with certain aspects of EMA guidance, such as inclusion of research projects in the initial protocol and developing a separate protocol for PASS, might have avoided some issues in TOSCA and hence should be considered in future rare disease patient registries.

The EMA guidance on Good Registry Practice offers valuable guidance for future registries and registry studies. These guidelines will also help harmonize the databases established across different registries in same disease areas. It is, however, to be noted that some of the expectations are simply not feasible in the context of rare diseases. For instance, collecting a very large number of variables open-endedly in a small population may be difficult owing to the burden on patients. Additionally, it cannot be expected that adequate financial means for open-ended registries with high data quality and completeness is available for each rare disease. The contribution of patient communities in rare disease, if properly engaged, can be instrumental to ensure high accrual and minimal loss to follow-up. Adopting additional measures to address the issues specific to rare disease registry is thus suggested for optimal outcomes.

## Author Contributions

RM performed the conceptualization and design of the manuscript, drafting, revising, final review, and provided approval of the manuscript to be published. HT performed the design of the manuscript, drafting, revising, final review, and provided approval of the manuscript to be published. JR performed the conceptualization and design of the manuscript, drafting, revising, final review, and provided approval of the manuscript to be published.

## Conflict of Interest

RM and HT are employees of Novartis. JR is Advisory Board and Lecturer for Abbvie, Novartis. JR is also Lecturer for Takeda, Merck Sharpe & Dohme, Grifols, and Leo. TOSCA was funded by Novartis Pharma AG. All authors approved the final version of the manuscript prior to submission.

## Abbreviations

AEs: adverse events
AHRQ: Agency for Healthcare Research and Quality
ATC: Anatomical Therapeutic Chemical
CTH: Clinical Trial Head
EBMT: European Society for Blood and Marrow Transplantation
ECFSPR: European Cystic Fibrosis Society Patient Registry
EMA: European Medicines Agency
ENCePP: European Network of Centers for Pharmacoepidemiology and Pharmacovigilance
EPIRARE: European Platform for Rare Disease Registries
EU: European Union
EU PAS: The European Union electronic Register of Post-Authorisation Studies
EUCERD: European Union Committee of Experts on Rare Diseases
EURD: European Union reference dates
GDPR: General Data Protection Regulation
GVP: Good Pharmacovigilance Practice
ICH: International Council for Harmonization
KOLs: key opinion leaders
MAH: Marketing Authorisation Holder
MedDRA: Medical Dictionary for Regulatory Activities
PAES: Post-Authorization Efficacy Study
PASS: Post-Authorization Safety Study
PIs: principal investigators
RCT: randomized controlled trial
RDs: rare diseases
RPs: research projects
RWD: real-world data
SAB: Scientific Advisory Board
SAP: statistical analysis plan
SEGA: subependymal giant cell astrocytoma
TAND: TSC-associated neuropsychiatric disorders
TOSCA: TuberOus SClerosis registry to increase disease Awareness
TSC: tuberous sclerosis complex
WC: working committee
WHO: World Health Organization.
